# MiR-29a-3p: a potential biomarker and therapeutic target in colorectal cancer

**DOI:** 10.1007/s12094-022-02978-6

**Published:** 2022-11-10

**Authors:** Wen-Yan Mo, Shi-Qiong Cao

**Affiliations:** Division of Gastroenterology, Liyuan Hospital, Tongji Medical College of Huazhong University of Science and Technology, Wuhan, 430077 Hubei China

**Keywords:** MiR-29a-3p, Colorectal cancer, Biomarker, Molecular mechanisms, Therapeutic target

## Abstract

Cancer is frequently caused by microRNAs, which control post-transcriptional levels of gene expression by binding to target mRNAs. MiR-29a-3p has recently been shown to play a twofold function in the majority of malignancies, including colorectal cancer (CRC), according to mounting evidence. Here, we not only briefly summarize such connection between miR-29a-3p and cancers, but aslo primarily evaluate the miR-29a-3p expression pattern, clinical applicability, and molecular mechanisms in CRC to provide a guide for future studies. This review established the diagnostic and prognostic value of miR-29a-3p abnormalty in a variety of clinical samples for CRC. Furthermore, current molecular mechanisms of miR-29a-3p for regulating cancerous biological processes such growth, invasion, metastasis, the epithelial-mesenchymal transformation process, and immunomodulation through its upstream regulatory factors and downstream targeted genes were briefly explored. More specifically, miR-29a-3p has been linked to a few medications that have been shown to have anticancer benefits. To sum up, miR-29a-3p is a promising biomarker and prospective therapeutic target for the diagnosis and prognosis of CRC, but further research is still needed to establish a theoretical basis for more practical applications.

## Introduction

Colorectal cancer (CRC) is the third most common cancer and the second most common cause of cancer worldwide [1]. Finding new biomarkers and medicines for early identification and efficient therapy is imperative due to the characteristics of CRC, which include strong concealment of clinical signs, a high degree of malignancy, and a dismal prognosis, particularly in the advanced stages [2, 3]. Thus, understanding the underlying mechanisms in the pathogenesis of CRC remains critical for developing new diagnostic or prognostic biomarkers and therapeutic approaches. As a heterogeneous disease, the carcinogenesis of CRC is a multifactorial and multistep process consisting of a sequential accumulation of environmental, genetic, and epigenetic factors [3]. MicroRNA dysregulation is an epigenetic alteration that has the potential to be a cancer-specific biomarker and a therapeutic target for CRC [4]. As a post-transcriptional regulator of gene expression, microRNAs are a class of highly conserved, endogenous, single-stranded, non-coding small RNAs composed of approximately 22 nucleotides [5]. Since these small RNA molecules are only expressed in certain types of cells and tissues, it has been shown that abnormal expression of miR-29a-3p plays a role in a number of pathophysiological processes, including liver fibrosis [6], cardiac remodelling [7], pulmonary vascular remodelling [8], fibro-inflammatory processes [9], neurodegenerative events [10]. More significantly, earlier studies have revealed the dual function of miR-29a-3p in a number of malignancies, particularly hepatocellular carcinoma (HCC) [45, 46]. Furthermore, on account of a growing body of studies, dysregulation of miR-29a-3p and increasing regulatory genes have been implicated in the tumorigenesis and development of various cancers via regulating tumor-related malignant biological and pathological processes such as proliferation, apoptosis, cell cycle, migration, invasion, angiogenesis, and epithelial-mesenchymal transformation (EMT) and so on. Of all the studies, most have reported that miR-29a-3p serves as a tumor suppressor; yet, existing evidence has also found it as an oncogene under certain circumstances. For example, miR-29a-3p was downregulated in head and neck squamous cell carcinoma [11], non-small cell lung cancer [109], and prostate cancer [12], but also upregulated in leukemia [13], and breast cancer [14]. Such miRNA has important clinical value for early diagnosis, targeted treatment, and improving the prognosis of various cancers [15]. In particular, in CRC, an increasing number of studies in vivo and in vitro experiments have demonstrated that miR-29a-3p dysregulation is positively or inversely correlated to the malignant biological processes through a multitude of regulatory mechanisms; however, the previous and the latest findings come to differ in such field. Correspondently, it’s necessary to sufficiently summarize the current findings of miR-29a-3p and its roles in diverse mechanisms, biological functions, and clinical value in CRC alone, and here we conducted a systematic review.

### Characteristic of MiR-29a-3p

**Fig. 1:**
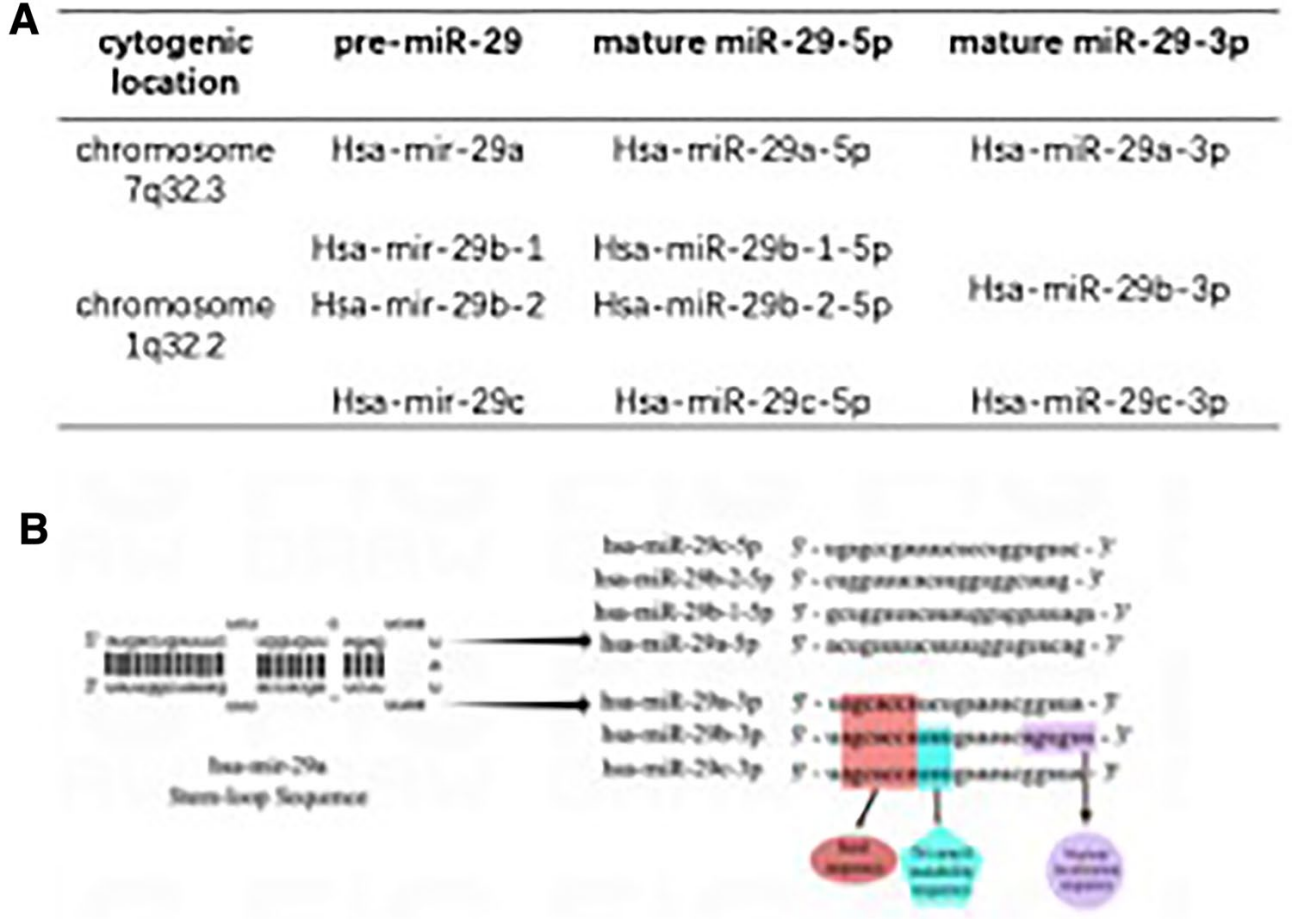
Schematic illustration of the miR-29a-3p family members focusing on location (**A**) and sequence (**B**). Seed sequence at position 2–8 nt (red box) is important for pairing with target messenger RNAs in the miR-29-3p family. Tri-uracil instability sequence at position 9–11 nt (blue box) presents in miR-29b-3p and miR-29c-3p. Nuclear localization sequence at position 18–23 nt (purple box) only exists in miR-29b-3p. Abbreviation: nt, nucleotide

**Fig. 2:**
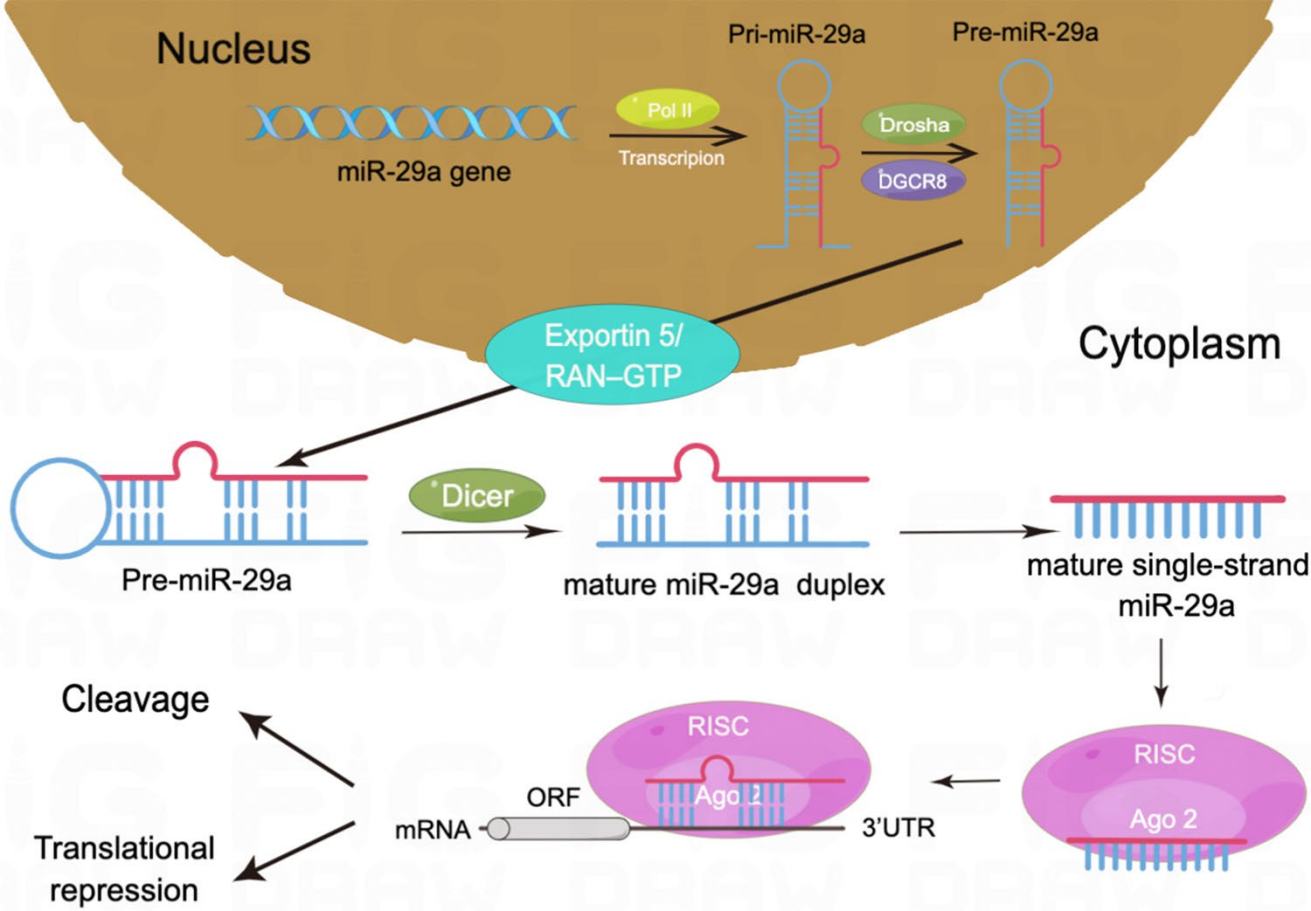
miR-29a-3p biogenesis. In the nucleus, RNA Polymerase II (Pol II) performs the initial transcription of miR-29a-3p to create a pri-miR-29a-3p transcript. Drosha, an RNase III enzyme, and DGCR8, a double-stranded RNA-binding protein, then convert the pri-miR-29a-3p transcript into pre-miR-29a-3p, an incomplete stem-loop RNA (64 nucleotides in length). Notably, pre-miR-29a-3p is exported into the cytoplasm by the RAN-GTP dependent transporter exportin 5, and processed by Dicer into miR-29a-3p duplex (a double-stranded miRNA of 22 nucleotides in length). The mature miR-29a-3p (a single-stranded miRNA of 22 nucleotides in length) is created once this dsRNA is unlocked. Mature miR-29a-3p binds to the 3'UTRs of mRNA targets and represses their production in two different ways after being integrated with Argonaute proteins into the RISC complex. This image was created using Figdraw.* Available online: https://www.figdraw.com.* *pri-miR-29a-3p* Primary miR-29a-3p, *pre-miR-29a-3p* precursor miR-29a-3p, *DGCR8* The double-stranded RNA-binding protein, *mRNA* Messenger RNA, *3’UTRs* 3’Untranslated regions, *RISC* MiRNA-induced silencing complex

According to reports, the human genome contains roughly 2300 miRNAs, which control more than 60% of protein-coding genes and represent 2–4% of all expressed genes [16, 17]. Discovered firstly in human Hela cells in 2001 [18], the miR-29 family comprises four species with identical seed sequences and two genomic clusters. While miR-29b-2/c is found in the miR-29c cluster, miR-29a/b-1 is found in the miR-29a cluster. The mature sequences of miR-29b-1 and miR-29b-2 are identical and there exists the same seed sequence but only a nucleotide difference outside the seed sequence among miR-29a, miR-29b, and miR-29c. The miR-29a/b-1 cluster and the miR-29b-2/c cluster are encoded on different chromosomes in the human genome, respectively, at positions 7q32.3 and 1q32.2 [19] (Fig. 1). MiR-29a-3p biogenesis, like that of other miRNAs, begins in the nucleus and then moves to the cytoplasm. A mature miRNA duplex is formed from pri-miRNA and pre-miRNA after they are transcribed by RNA polymerase II and cleaved primarily by the RNase III enzymes Drosha and Dicer. MiR-29a-3p or 5p is selected from the 5′ or 3′ arm of the pre-miRNA with an imperfect stem-loop structure and then loaded onto Argonaut proteins to yield RNA-induced silencing complex (RISC), which can specifically bind to the perfect or imperfect complementary sites in the 3′untranslated region (3'UTR) of messenger RNAs, thus regulating downstream gene expression at the post-transcriptional level by degradation or translational repression of the target [5, 20] (Fig. 2). Although complex mechanisms may influence miRNA arm selection, 3p arm miRNAs are the most abundant in the miR-29 family in humans [21]. Accordingly, the majority of studies related to colorectal cancer focus on miR-29a-3p rather than miR-29a-5p as well. Despite being directly equivalent to miR-29a in previous studies,miR-29a-3p is now considered a more standardized naming for its highest expression abundance. It is worth noting that a single miRNA can target several target genes. In the meanwhile, one gene can also be regulated by multiple miRNAs. As a result, miR-29a-3p and the downstream genes that modulate its functions form a complex network.

### MiR-29a-3p as a biomarker in CRC

#### MiR-29a-3p in tissues as a malignant marker

Until now, consistency and reproducibility of research have been major barriers to making miR-29a-3p in tissues a convincing potential malignant marker for CRC diagnosis and prognosis prediction (Table 1). Clearly, a substantial body of evidence has revealed that the differential expression of miR-29a-3p in CRC tissues was up-regulated when compared to adjacent normal tissues or healthy patients [22–24]. It is worth noting that the levels of miR-29a-3p were significantly higher in colorectal cancer tissues compared to normal tissues, but not in adenomas [25]. According to the TNM staging system, He et al. found that miR-29a-3p expression levels were lower in stage I CRC patients than in stage II and III [26]. Weissmann-Brenner et al. obtained comparable results and went on to conduct a ROC analysis. Up-regulated miR-29a-3p in tissues had a 94% positive predictive value and a 67% sensitivity for distinguishing CRC patients without recurrence. This work also found that miR-29a-3p levels in tissues were positively related to disease-free survival, and were confirmed as an independent prognostic factor for patients with II-stage CRC by multivariate analysis [27]. In addition, Krbal et al. proved that higher expressed miR-29a-3p was found in isolated colon cancer (CC) cells from clinical specimens compared with CC-derived lymph node metastatic cells [28].

In contrast to the clinical finding above, a couple of studies have indicated lower expression of miR-29a-3p could lead to poorer tumor progression. For example, Wang et al. demonstrated that the expression of miR-29a-3p in tissues was inversely related to TNM staging and lymph node metastasis of CRC [29]. Additionally, there was no statistical significance among miR-29a-3p expression and other clinical parameters, including differentiation and distant metastasis. Furthermore, Kuo et al. showed that CRC patients with early recurrence had lower miR-29a-3p expression levels than those without early recurrence, and in return lower miR-29a-3p levels were positively associated with shorter one-year survival rates [30]. Taken together, the expression pattern of miR-29a-3p in CRC is still debatable, despite the fact that it may be a marker of malignancy in CRC with some clinical relevance. The disagreement is probably given rise to the small sample size, criteria for inclusion and exclusion, individual characterization, analytical procedures, and so on.

**Table 1:** miR-29a-3p dysregulation in tissues and its clinical implications in CRC

Clinical sample (CRC, controls)	Patients	Expression	Country/Year	Clinical implication	References
Tissues	54/42 (CRC/HC)	↑	Turkey/2015	–	[22]
Tissues, Serum (stage III)	12/12 (CRC/NPT) 30/26 (CRC/HC)	↑	Spain/2013	–	[23]
Tissues	82/82 (CRC)	↑	China/2014	Higher risk of metastasis and poorer overall survival	[24]
Tissues	19/20 (CRC/HC)	↑	Japan/2016	Only dysregulation in CRC, not in adenomas	[25]
Tissues	28/28 (CRC/NPT)	↑	Malaysia/2017	More advanced CRC staging	[26]
Tissues (stage I and II)	59/59 (CRC/NPT)	↑	Israel/2012	Sen 67%; PPV 94% for diagnosis; Longer disease-free survival in stage II	[27]
4 isolated primary colon cancer cells and its lymph node metastatic cells	-	↑	Czech/2016	Higher in isolated colon cancer cells than CC-derived lymph node metastatic cells	[28]
Tissues	80/80 (CRC/NPT)	↓	China/2021	Higher risk of advanced TNM staging and lymph node metastasis	[29]
Tissues	78/79 (CRC/NPT)	↓	China/2012	Higher risk of early recurrence and lower 1-year survival rate	[30]

↑ up-regulation, ↓ down-regulation

*AA* Advanced adenomas, *CC* Colon cancer, *CRC* Colorectal cancer, *HC* Health controls, *NPT* Normal precancerous tissues from CRC patients

### Circulating MiR-29a-3p as a biomarker

Emerging evidence elucidated that miR-29a-3p can act as potential non-invasive diagnostic biomarkers in CRC; they are measurable in various body fluids such as serum, plasma, and exosome in sera, but among those were only a few studies as follows focusing on precancerous lesions and early-staged CRC to prevent CRC, which are curable and then function to reduce high morbidity and mortality of CRC (Table 2). In the serum samples, using two phases for screening and validation, Yamada et al. made a comparison of miR-29a-3p levels between the patients with precancerous lesions in colorectal tissues and health controls. This study found that the group of precancerous lesions expressed much higher levels of serum miR-29a-3p with an AUC of roughly 0.74 and the subgroup of advanced neoplasia (AN) was almost the same. Furthermore, this work found that serum miR-29a-3p levels were inversely related to the size of colorectal lesions. Last but not at least, in terms of combined biomarkers in the peripheral blood, the AUC values for the combination of the serum miR-21, miR-29a-3p, and miR-125b in discriminating precancerous lesions and AN patients from healthy controls were approximately 0.83 and 0.76, respectively [31]. Later, Uratani et al. used small samples and reported that serum miR-29a-3p alone had a higher AUC of 0.85 for advanced adenomas, although the AUC of 0.68 was lower for all adenomatous polyps. Additionally, the miR-29a-3p levels in exosomes did not show a favorable diagnostic lower at all [32]. Furthermore, Marcuello et al. found that the diagnostic value of the other serum miRNAs panel showed an improvement in differentiating advanced adenomas (AA) from healthy controls. The combination of six miRNAs (miR-19a-3p, miR-19b-3p, miR-335-5p, miR-29a-3p, miR-15b-5p, miR-18a-5p) yielded a higher AUC of 0.80 with 81% sensitivity, and 63% specificity [33]. In the plasma samples, Huang et al. came to a similar conclusion and reported that the high levels of miR-29a-3p alone could be used to discriminate AA from healthy controls with an AUC of 0.77, with 62% sensitivity and 85% specificity. Additionally, combined with miR-92a could enhance the sensitivity up to 73% [34]. However, another case–control study in Spain reported that the statistical significance of elevated plasma miR-29a-3p levels in AA wasn’t found [35]. Therefore, the robustness remains unclear for different circulating samples in diagnosing AA, and much more samples in different countries and specimen sources are needed to be validated.

**Table 2:** miR-29a-3p dysregulation in different body fluids and its clinical implications in CRC

Clinical sample	Patients	Expression	Country/Year	Cut-off	Diagnostic values	Prognostic values	References
Precancerous lesions							
Serum	128/52 (PL/HC)	↑	Japan 2015	-	1. AUC, 0.74; 2. & 2 miRs, AUC, 0.83	–	[31]
Serum	(AN/HC)	↑	Japan 2015	-	1. AUC, 0.73 2.miRs, AUC, 0.76	–	[31]
Serum	26/44 (PL/HC)	↑	Japan 2016	-	AUC, 0.68; Sen, 72%; Spec, 66%;	–	[32]
Serum Exosome	7/44 (AA/HC)	↑	Japan 2016	-	1.(Serum)AUC, 0.85 2.(Exosome)AUC, 0.53	–	[32]
Serum	74/80 (AA/HC)	↑	Spain 2019	-	& 5 miRs, AUC, 0.80; Sen, 81%; Spec, 63%	–	[33]
Plasma	37/39 (AA/HC)	↑	China 2010	1.21	1. AUC, 0.77; Sen, 62%; Spec, 85%; 2. & mR-92a, AUC, 0.77; Sen, 73%; Spec, 80%	–	[34]
Plasma	40/53 (AA/HC)	–	Spain 2013	–	Significance not found	–	[35]
CRC							
Plasma	100/59 (CRC/HC)	↑	China 2010	1.33	1. AUC, 0.84; Sen, 69%; Spec, 89%; 2. & mR-92a, AUC, 0.88; Sen, 83%; Spec, 85%	–	[34]
Plasma	42/53 (CRC/HC)	↑	Spain 2013	–	–	–	[35]
Serum	136/52 (CRC/HC)	↑	Japan 2015	–	Significance not found	–	[31]
Serum	85/78 (CRC/HC)	↑	China 2018	5.02	1. AUC, 0.88; Sen, 69%; Spec, 95%; 2. & 3 miRs, AUC, 0.95; Sen, 85%; Spec, 99%;	–	[38]
Serum	59/80 (CRC/HC)	↑	Spain 2019	-	1. & 5 miRs, AUC, 0.74; Sen, 81%; Spec, 56% 2. & 5 miRs and fecal HIT, AUC, 0.88; Sen, 81%; Spec, 78%	–	[33]
Serum	73/30 (CRC/C)	↑	China 2017	-	–	Predict Liver metastasis: 1.AUC, 0.85; Sen, 63%; Spec, 84%; 2. & COX-2 +MMP2, AUC, 0.90; Sen, 71%; Spec, 95%	[40]
Plasma	122/0 (CRC)	↑	America	-	–	Predict 3-year recurrence: AUC, 0.70	[41]
Extracellular vesicles	18/73 (CRC/HC)	↑ (non-EV/EV ratio)	China	-	AUC, 0.70	–	[42]
Saliva	51/37 (CRC/HC)	↑	Spain	0.51 (for miRs)	1.AUC, 0.63; Sen, %; Spec, %; 2. & 4 miRs, AUC, 0.75; Sen, 70%; Spec, 45%; 3. & 4 miRs and CEA, Sen, 89%	Poorer progression-free survival	[43]
Feces	80/51 (CRC/HC)	↓	China	-	1.colon < rectum 2.AUC, 0.78; Sen, 85%; Spec, 61%;	–	[44]

*PL Precancerous lesions, HC* Healthy controls, *C* Controls including patients with non-malignant diseases

↑ Up-regulation, ↓ Down-regulation

As for CRC diagnosis, circulating miR-29a-3p is so stable that it acts as a promising non-invasive biomarker while colonoscopy as the gold standard is invasive, troublesome, and relatively expensive, and the widely used stool/blood-based tests are of low diagnostic power [36, 37]. At first, Huang et al. studied the miR-29a-3p levels for CRC screening and detection. Based on the plasma specimens, they found that compared with healthy controls, miR-29a-3p levels were highly expressed in the CRC group in China [34], and later, a study carried out a study in Spain came to the same conclusion [35]. In addition, Huang et al. also evaluated the diagnostic value of miR-29a-3p alone for CRC with an AUC of 0.84, 69% sensitivity, and 89% specificity. Furthermore, combined with miR-92a showed improvement in the diagnostic value for CRC with an AUC of 0.88, a sensitivity of 83%, and a specificity of 85%. However, there was no statistical significance between plasma miR-29a-3p level and TNM staging, tumor location, and other clinical parameters [34]. Different from the specimens in plasma, Yamada et al. reported that miR-29a-3p levels in sera were higher in CRC patients than in controls, but no significant difference was found, which was inconsistent with the previous and subsequent studies [31]. In the last five years, both miR-29a-3p alone and a combination of other miRNAs in sera have shown considerable diagnostic values. Liu et al. demonstrated that serum miR-29a-3p level was significantly higher in CRC patients than in healthy controls and showed a higher AUC of 0.88 with 69% sensitivity and 95% specificity, compared with the AUC of 0.808 and 0.705, respectively, for serum CEA and CA19-9. This work further found the optimal combination of miRNAs in serum for CRC well-diagnosis and finally reported that high levels of the 4-miRNAs panel (miR-21, miR-29a-3p, miR-92a, miR-125b) for discriminating CRC yielded the highest diagnostic value with an AUC value of 0.95, a sensitivity of 85% and a specificity of 99% than any miRNAs reported ever before [38]. Concerning Marcuello M's research, whether a serum 6-miRNAs panel was alone or in combination with hemoglobin concentration in feces, their diagnostic values for CRC in diagnosing CRC were inferior to the previous result [33]. Above all, although there seems to be a lack of consistency in different circulating samples and the diagnostic values were discrete, miR-29a-3p is a promising biomarker for CRC and further studies are worthwhile.

Notably, El-Daly et al. constructed a murine model of colitis-related CRC to initially access the dynamic changing in the expression level of miR-29a-3p during the stepwise carcinogenesis process and the consistency of miR-29a-3p level in serum and corresponding tissue samples. Noteworthy, higher miR-29a-3p levels were found in the advanced stage of carcinogenesis and yielded higher diagnostic values for early colon lesions than serum CEA and CA-19-9. Furthermore, this work confirmed that serum miR-29a-3p may derive from tissue for its consistency in the diagnostic power during the carcinogenesis process. However, only vivo experiments in the mouse model could limit the clinical application of the above results, and further clinical correlations are still needed to be done [39].

Although not much, some studies were performed to evaluate the circulating miR-29a-3p levels to predict the progress and prognosis of CRC. In general, higher circulating miR-29a-3p levels showed a higher risk of malignant progression and poor prognosis. For example, Zhou et al. revealed that upregulation of miR-29a-3p in sera could predict the liver metastasis of CRC with a high AUC of 0.85, 66% sensitivity, and 84% specificity. Further combination with serum COX-2 and matrix metallopeptidase 2(MMP2) show higher diagnostic values of AUC = 0.90, 72% sensitivity, and 95% specificity [40]. Yuan et al. found that a high preoperative miR-29a-3p level in plasma could predict relapse of CRC within 3 years with an AUC of 0.70 while dynamic postoperative plasma miR-29a-3p levels did not correlate with CRC recurrence [41]. Interestingly, to access and analyze the non-vesicular to extracellular vesicles miR-29a-3p expression ratio in serum for the first time, Shiosaki et al. enrolled a cohort of CRC patients from different races from Japan and Hawaii. However, they found that compared with healthy controls, such ratio was higher in both the early-staged (stage I, II) and advanced (stage III, IV) CRC patients, and the AUC for diagnosis of CRC was 0.70. Additionally, the levels of miR-29a-3p only in extracellular vesicles showed a stepwise reduction during the progression of CRC stages, which could explain the corresponding increased ratio [42].

#### Fecal and salivary MiR-29a-3p as a biomarker

Although these sample types seem to be more non-invasive and easier to get than blood, there indeed exists controversy in the expression of miR-29a-3p in between saliva and feces among the limited literature that has been published (Table 2). Rapado-González et al. have proven that salivary miR-29a-3p levels were significantly higher in CRC patients than in healthy controls, and yielded an AUC value of 0.63 to differentiate CRC from normality. Furthermore, a 5-miRNAs panel (miR-186-5p, miR-29a-3p, miR-29c-3p, miR-766-3p, miR-491-5p) yielded a higher AUC value of 0.75, with a sensitivity of 70%, compared with a sensitivity of 45% for serum CEA. Notably, this 5-miRNAs panel combined with serum CEA could enhance a higher sensitivity up to 89%. In terms of demographic and clinicopathological characteristics in CRC patients, the salivary miR-29a-3p levels were only significantly higher in males than in women, however, no significant association was found between salivary miR-29a-3p levels and other parameters. Additionally, the study also confirmed that these salivary 5-miRNAs panel levels are independent prognostic factors for progression-free survival in patients with IV CRC by univariate and multivariate cox regression analysis [43].

Contrary to Rapado-González ‘s findings, Zhu et al. showed that compared with healthy controls, fecal miR-29a-3p was abnormally downregulated in stool samples of CRC patients with an AUC of 0.78, 85% sensitivity, and 61% specificity, which surpassed the partial diagnostic values of miR-223 (AUC: 0.744, 60% sensitivity), and miR-224 (AUC: 0.745, 75% sensitivity). Different from all the above studies, a significant association was found between fecal miR-29a-3p level and location of CRC, that is, colon cancer expressed a lower level of miR-29a-3p than rectal cancer [44]. In conclusion, samples were not enough to clarify the contradiction in miR-29a-3p dysregulation and clinical values in different sources of samples in CRC. What’s more, multicenter researches, unified methodology, and calculation methods are also further needed.

### The mechanisms of MiR-29a-3p in cancers

As was already ascertained, miR-29a-3p regulates a variety of malignancies due to its dual expression patterns. The underlying molecular mechanism and functional role of miR-29a-3p, which functions as a frequently downregulated tumor suppressor or occasionally an oncogene in a variety of cancers through various upstream genes and target genes, have been the subject of an increasing number of studies over the past three years. Consistently, given the information that is now available, a sufficient summary of miR-29a-3p’s dual roles in HCC was provided last year [45]. Therefore, Table 3 details the most current discoveries about the roles and mechanisms of miR-29a-3p and its validated gene in cancer. In particular, considering the preceding review [46], we partially concentrate on the research connecting miR-29a-3p and competing endogenous RNAs in malignancies, which may, to some extent, enhance the activity of miR-29a-3p alone but has previously been constrained.

**Table 3:** The functions and proven mechanisms of miR-29a-3p in the biological processes of cancers

Cancers	Upstream gene	Target genes	Biological processes	References
miR-29a-3p as a tumor suppressor				
LC	LncRNA NEAT1	–	Proliferation, metastasis	[74]
	–	STAT3	Proliferation, migration, invasion	[56]
PTC	–	DPP4	Proliferation, migration, invasion	[47]
OSCC	lncRNA SNHG20	DIXDC1/Wnt	Proliferation, migration, invasion	[75]
GC		KDM5B1	Proliferation	[48]
	lncRNA NR2F1-AS1	VAMP7	Proliferation, migration, invasion	[67]
	Circ29	VEGF	Proliferation, migration, angiogenesis	[57]
	LncRNA LIFR-AS1	COL1A2	Proliferation, apoptosis, migration, invasion	[53]
	HOXA-AS3	LTβR/NF-κB	Proliferation, migration, invasion	[62]
PDAC	MYC	LOXL2	Metastasis, EMT process	[59]
HCC	–	HIF-1α、ANGPT2	Carcinogenesis	[76]
	–	LOX、VEGFA	Proliferation, apoptosis, migration, invasion	[58]
CCA	LncRNA TUG1	–	Proliferation, apoptosis, migration, invasion	[77]
NSCC	–	REV3L	Cisplatin sensitivity	[78]
LUAD	–	C1QTNF6	Proliferation, migration, invasion	[61]
LACC	–	AKT2	Proliferation	[49]
BC	lncRNA DUXAP8	SAPCD2	Proliferation, apoptosis	[50]
	circPVT1	AGR2/HIF-1α	Proliferation, apoptosis, migration, invasion,	[79]
EOC	LncRNA HCG18	TRAF4/5	Proliferation, migration, invasion, EMT process, proinflammation	[80]
CC	–	DNMT3A、DNMT3B	Proliferation, cell cycle arrest	[64]
	–	SNIP1	Proliferation, migration	[81]
	–	DNMT1/SOCS1/NF-κB	Proliferation, apoptosis, migration, invasion	[65]
	–	SIRT1	Metastasis, EMT process	[82]
EC	–	VEGFA/CDC42/PAK1	Proliferation, migration, invasion	[83]
	–	DNMT3B/SOCS1/NF-κB	Apoptosis, migration, invasion, EMT process	[66]
OC	lncRNA DUXAP8	–	Proliferation, metastasis	[84]
	circKRT7	COL1A1	Growth, migration, invasion	[54]
	CircACAP2	COL5A1	Proliferation, migration, invasion	[55]
PC	circFOXO3	SLC25A15	Apoptosis, Cell cycle arrest	[12]
	Circ_0044516	–	Proliferation, metastasis	[85]
AML	lncRNA XIST	MYC	Proliferation, apoptosis, drug sensitivity	[86]
	LncRNA-H19	Wnt/β-catenin pathway	Proliferation, apoptosis	[68]
	–	OTUB2/TRAF6/NF-κB	Proliferation, migration, invasion	[63]
OS		IGF1	Proliferation, migration, invasion; autophagy	[87]
	LncRNA H19	LASP1	Proliferation, migration, invasion;	[69]
	LncRNA LIFR-AS1	NFIA	Proliferation, invasion, apoptosis	[88]
Glioma	–	ROBO1	Invasion, angiogenesis	[60]
GBM	–	PDGFC、PDGFA	Growth, apoptosis, migration, invasion,	[52]
	P53	MDM2	Proliferation, migration, invasion, apoptosis, drug Sensitivity	[51]
RMS	–	GEFT	Proliferation, apoptosis, migration, invasion	[89]
miR-29a-3p as an oncogene				
RCC	circFOXO3	–	NK cells cytotoxicity	[70]
OSCC	–	SOCS1/STAT6	M2 subtype macrophage polarization	[71]
OC	–	FOXO3/AKT/GSK3β	Immune escape	[90]
AB	–	CTNNBIP1	Proliferation, migration, invasion	[72]
BC	–	PC4	Growth, migration, invasion, EMT process	[91]
BCSC	–	SUV420H2/H4K20/ CTGF/EGR1	Migration, invasion	[73]

*AB* Ameloblastoma, *AML* Acute myeloid leukemia, *BC* Breast cancer, *BCSC* Breast cancer stem cells, *CCA* Cholangiocarcinoma, *CC* Cervical cancer, *EC* Endometrial cancer, *EOC* Epithelial ovarian cancer, *GBM* Glioblastoma, *GC* Gastric cancer, *PDAC* Pancreatic ductal adenocarcinoma, *HCC* Hepatocellular carcinoma, *LC* Laryngeal carcinoma, *LACC* Lacrimal gland adenoid cystic carcinoma, *LSCC* Laryngeal squamous cell carcinoma, *LUAD* Lung adenocarcinoma, *NSCC* Non‑small cell lung cancer, *OS* Osteosarcoma, *OSCC* Oral squamous cell carcinoma, *OC* Ovarian cancer, *PTC* Papillary thyroid cancer, *PC* Prostate RCC, Renal cell carcinoma, *RMS* Rhabdomyosarcoma

MiR-29a-3p functions as a tumor suppressor by preventing cell proliferation, invasion, migration, cell cycle, EMT process, angiogenesis, drug resistance, and enhancing apoptosis in various cancers. This is achieved by binding to the 3′UTR of validated target genes of all types. Numerous oncogenes targeted by miR-29a-3p were experimentally confirmed to be down-regulated in terms of inhibiting tumor growth, including DPP4 [47], KDM5B1 [48], AKT2 [49], SPAPCD2 [50], MDM2 [51], PDGF [52], and others. The direct targets of miR-29a-3p, such as the associated genes collagens (COL) [54–55], STAT3 [56], VEGF [57], LOX [58], LOXL2 [59], ROBO1 [60], mostly affected the tumor microenvironment, which was the site of angiogenesis, EMT process, and metastasis. In addition, miR-29a-3p-targeted genes C1QTNF [61], LTβR [62], and OTUB2 [63] that are involved in immunomodulation in malignancies were also confirmed to contribute to tumor growth and progression. With regard to cervical cancer (CC) [64, 65] and endometrial cancer (EC) [66], numerous pieces of evidence revealed that miR-29a-3p played a detrimental role in the growth of tumors by targeting epigenetic modifiers such DNMT1, DNMT3A, and DNMT3B. The cytokine signaling 1 (SOCS1) gene was consistently directly demethylated by these epigenetic modifiers, which in turn controlled the NF-kB signaling pathway in both the CC and EC [65, 66].

More recent studies have shown that miR-29a-3p expression levels are negatively regulated by competing endogenous RNAs, such as long non-coding RNAs (lncRNA) and circular RNAs (circRNA), through the miR-sponging process. For instance, by competitively sponging miR-29a-3p, lncRNA HOXA-AS3, lncRNA LIFR-AS1, lncRNA NR2F1-AS1, and circ29 have a promotive function in the carcinogenesis of gastric cancer [62, 53, 57, 67]. Importantly, Li et al. identified one of them, circ29, as a miR-29a-3p sponge that supported tumor angiogenesis [57]. In addition to being recognized as one of the ceRNAs in HCC [45], lncRNA TUG1 was also recognized as a potential anti-cancer factor due to its direct inhibition of miR-29a-3p production in cholangiocarcinoma. Furthermore, lncRNA H19 sponged and downregulated miR-29a-3p in both acute myeloid leukemia (AML) and osteosarcoma, which prevented cell death and enhanced cell proliferation, migration, and invasion [68, 69]. Interestingly, in prostate cancer (PC), Kong et al. proved that circFOXO3 exerted a tumor-promoting role in inhibiting the capacity of cell apoptosis and cell cycle arrest via directly down-regulating miR-29a-3p, and then up-regulating SLC25A15 [12]. Inversely, according to Yang et al., although circFOXO3 overexpression was observed in renal cell carcinoma (RCC), it served as an inhibitor in vitro by directly sponging miR-29a-3p, which increased NK cell cytotoxicity toward RCC cells. On the other hand, miR-29a-3p overexpression weakened this immunomodulation process [70].

Several pieces of evidence have shown that miR-29a-3p works as an oncogene in many different types of malignancies, including RCC, oral squamous cell carcinoma (OSCC), ovarian cancer (OC), breast cancer, and ameloblastoma, in contrast to the tumor-suppressive role that has been discussed in cancers. As previously reported, circFOXO3 demonstrated the opposite impact through tumor immunomodulation and tumorigenicity in several malignancies. In this regard, it was established that circFOXO3 directly binds to and inversely modulatesmiR-29a-3p. In the tumor immune microenvironment, miR-29a-3p acted to attenuate the NK cell cytotoxicity towards RCC cells augmented by circFOXO3, which made it a tumor-promoter [70]. Furthermore, Lu et al. indicated that tumor-associated macrophages (TAMs)-derived miR-29a-3p in exosome could foster OC cell proliferation in vitro [90]. Mechanistically, miR-29a-3p functioned as a tumor-promoting factor by downregulating the FOXO3-AKT/GSK3β axis and subsequently suppressing the expression of PD-L1, which was a known target molecule for anti-tumor immunotherapy, to shield OC cells from immune escape. Additionally, Cai et al. showed thatmiR-29a-3p was strongly expressed in the tissues of OSCC and encouraged M2 subtype macrophage polarization in the form of OSCC-derived exosomes produced by OSCC, which increased cell proliferation, invasion in vitro, and tumor growth in vivo. The mechanism was that miR-29a-3p bound to SOCS1 and further up-regulated transcriptional activator 6 (STAT6) in macrophages [71]. Liu et al. found that miR-29a-3p levels in tissues were higher than normal in ameloblastoma. Additional research showed that miR-29a-3p directly targets the β-catenin interacting protein 1 (CTNNBIP1), which negatively regulated the activity of the Wnt/-βcatenin pathway and facilitates cell migration and invasion [72]. Sikder et al. discovered PC4, a transcriptional coactivator, as a target of miR-29a-3p in breast cancer, which led to tumor growth both in vitro and in vivo. In the same year, Wu et al. found that the overexpression of miR-29a-3p in breast cancer cells facilitated both in vitro and in vivo migration and invasion by binding to SUV420H2, a histone methyltransferase that preferentially trimethylates Lys-20 of histone H4 (H4K20). H4K20 trimethylation was further modulated by connective tissue growth factor (CTGF) and growth response protein-1 (EGR1), which are involved in the EMT process [73].

### The mechanism of MiR-29a-3p in CRC

#### MiR-29a-3p as an oncogene

**Fig. 3:**
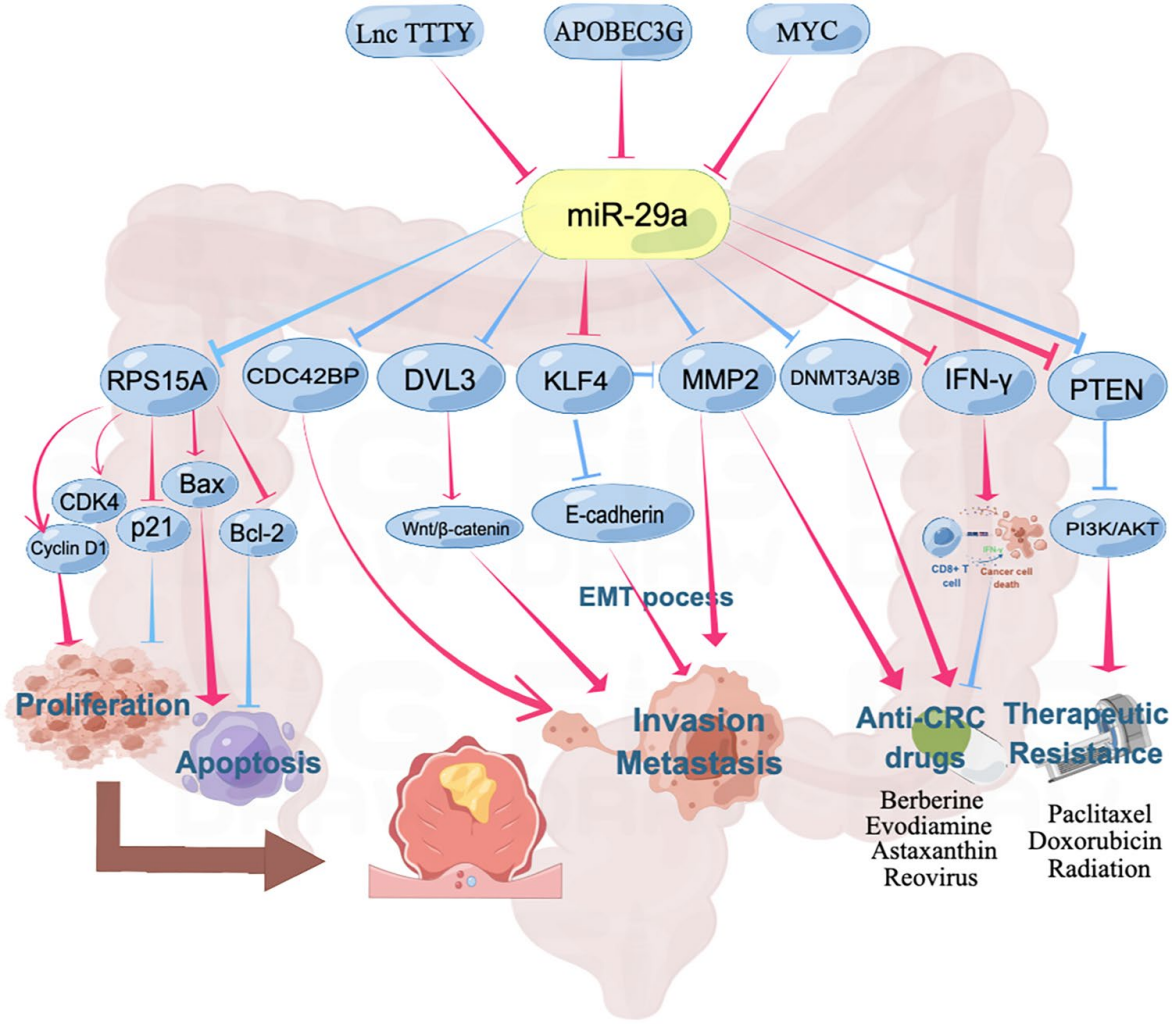
miR-29a-3p-related downstream and upstream genes and biological functions in CRC

Despite the considerable clinical studies described above showing that CRC clinical samples express abnormally high levels of miR-29a-3p, there has been little research on the mechanism underlying miR-29a-3p’s role as an oncogene in CRC (Fig. 3). Mechanistically, miR-29a-3p promoted invasion, metastasis, and drug resistance in CRC by directly targeting KLF4 and PTEN. The normal gastrointestinal tract has been shown to express KLF4, a transcription factor with zinc finger structure and a member of the KLF family. The normal gastrointestinal tract has been shown to express KLF4, a transcription factor with zinc finger structure and a member of the KLF family [110]. KLF4 was abnormally down-regulated in colorectal adenoma and CRC, respectively, and was involved in the development of CRC, according to earlier investigations [93, 94]. According to research by Tang et al., overexpressed miR-29a-3p increased invasion and metastasis as well as the EMT process in vitro by directly binding to KLF4 at both the mRNA and protein levels. It also affected the expression of MMP2 and E-cadherin. On the other side, the promotive role of miR-29a-3p in CRC could be reversed by the restoration of KLF expression. Additionally, in vivo experiments using a mouse model of CRC treated with miR-29a-3p revealed a dramatically reduced level of KLF4 expression in the liver-metastatic nodules. As previously noted, miR-29a-3p was expressed at higher levels in CRC tissues than in healthy controls, which increased the risk of metastasis and decreased overall survival in CRC patients [24]. In terms of chemotherapeutic resistance, paclitaxel-resistant SW480 cells had greater levels of miR-29a-3p than did non-resistant SW480 cells, and miR-29a-3p was linked to acquired paclitaxel resistance via inhibiting the PTEN/PI3K/AKT pathway. Additional in vitro studies supported the finding that transfection of the miR-29a-3p inhibitor had an inhibitory effect on cell growth and caused apoptosis in CRC via increasing PTEN expression [95]. Regarding radiotherapy resistance, both malignant and normal cells, such as human intestinal epithelial crypt cell (HIEC), HT29, HCT116, and DLD-1 cells, showed increased miR-29a-3p levels following radiation treatment. Interestingly, it's noted that the molecular mechanism of radiation resistance caused by miR-29a-3p overexpression was the same as that reported above by LL et al. [96].

#### MiR-29a-3p as a tumor suppressor

A few other mechanistic studies have demonstrated that miR-29a-3p acts as a tumor suppressor and inhibits the growth, invasion, and metastasis of CRC cells through a number of target genes, in contrast to the aforementioned clinical or experimental studies that discovered the cancer-promoting effects of miR-29a-3p (Fig. 3). Zheng et al. provided evidence that miR-29a-3p expression was abnormally down-regulated in CRC tissues and related CRC cell lines, including DLD-1, RKO, SW480, and HCT116 cells. Additionally, miR-29a-3p overexpression reduced the growth of cancer cells, prevented the progression of the cell cycle into the G0/G1 phase, and brought about apoptosis. More in vitro analysis confirmed that RPS15A was a direct target of miR-29a-3p. Overexpression of RPS15A, a ribosomal protein involved in ribosomal assembly and translation, had the opposite impact of miR-29a-3p’s anti-CRC by partially down-regulating the expression of p21 and Bcl-2 and up-regulating the expression of CDK4, cyclin D1, and Bax [97]. Additionally, Disheveled family member DVL3 plays a carcinogenic role in many tumors by acting as a receptor and downstream target of cytoplasmic scaffold proteins through the activation of Wnt and NOTCH signaling pathways [98]. In a more recent study, Zheng et al. asserted that miR-29a-3p inhibited the expression of DVL3 at the mRNA and protein levels in HT29 and HT-116 cells, functioning to prevent cell growth and metastasis and reduce apoptosis. Furthermore, in CRC tissues and paired neighboring normal tissues, an inverse connection was detected between miR-29a-3p and DVL3 protein levels. Additionally, it was shown that the long non-coding RNA TTTY15 acts as a negative upstream regulator of miR-29a-3p [99]. It is well known that CDC42BP, a member of the serine protein kinase family, promotes tumor invasion and migration via modulating actin [100]. In vitro research by He et al. revealed that miR-29a-3p overexpression inhibited cell invasion and migration in CRC. Up-regulation of cytoskeletal regulatory genes such as CDC42BP, IQGAP2, and SSH1 was detected at the mRNA and protein expression levels following the suppression of miR-29a-3p. Knockdown of CDC42BP in vitro partially abrogated the anti-CRC effect generated by miR-29a-3p, and this target was predicted as a downstream target of miR-29a-3p by public databases; nevertheless, the binding link between these two genes lacked further experimental verification [26]. Besides, in a mouse model of a malignant colonic tumor generated by TGF-β, Huang et al. discovered that the expression levels of miR-29a-3p and its anticipated target genes DNMT3A/3B were considerably down-regulated. However, a related experiment to confirm the interaction between DNMT3A/3B and miR-29a-3p was omitted. In addition, it appeared that miR-29a-3p and DNA methyltransferases formed a negative feedback loop. The levels of miR-29a-3p were partially re-expressed under treatment with a DNMT inhibitor, though they remained lower than the normal controls [101]. Similarly, Gerovska et al. have developed a mouse model of CRC liver metastasis and found that miR-29a-3p expression was significantly reduced in the liver tumor microenvironment cells compared to the healthy controls. Microarray analysis suggested a link between histone modification and DNA methylation [102]. Besides that, APOBEC3G, one of the metastasis-related genes, was also shown by Ding et al. to facilitate cell invasion and metastasis in colon cancer cells using an orthotopic animal model of CRC. Regarding the mechanism, they confirmed in vitro using PCR array that APOBEC3G reduced miR-29a-3p levels, leading to elevated expression of matrix metalloproteinase 2(MMP2) [103].

Lnc TTTY and two oncogenes, MYC and APOBEGC3G could inhibit miR-29a-3p. miR-29a-3p directly targeted RPS15A, MMP2, DVL3, CDC42BP, DNMT3A/3B, and PTEN to impede malignant biological processes. However, miR-29a-3p boosted malignant biological processes by binding to KLF4, IFN-γ, and PTEN. Additionally, only Wnt/β-catenin and PI3K/AKT signaling pathways are verified in CRC. Consequently, miR-29a-3p could promote or suppress the malignant biological functions in CRC, such as cell proliferation, apoptosis, invasion, metastasis, tumor growth, and therapeutic resistance. This figure was created with Figdraw. Available online: https://www.figdraw.com. (The red lines emitted indicate carcinogenic factors and the blue ones indicate suppressive factors.)

### Mir-29a-3p-based therapy for CRC

#### MiR-29a-3p in chemoradiotherapy resistance

Based on the results of the available experimental research, aberrant expression of miR-29a-3p can either increase or decrease resistance to radiation and chemotherapy by inhibiting the tumor suppressor PTEN (Fig. 3). For chemotherapy resistance, higher amounts of miR-29a-3p were discovered in paclitaxel-resistant SW480 cells than in SW480 cells, according to research by LL et al. This indirectly activates the PI3K/AKT signaling pathway by binding to PTEN 3'UTR. MiR-29a-3p inhibitor in vitro enhanced PTEN expression to reduce CRC cell proliferation and induce apoptosis [95]. In contrast to this observation, Shi X et al. showed that miR-29a-3p overexpression in CRC cells played a detrimental impact in doxorubicin (DOX) resistance by directly binding to PTEN and reducing PTEN expression. In particular, HT29/DOX doxorubicin-resistant cancer cells expressed less miR-29a-3p than HT2 cells did. MiR-29a-3p mimic transfection further decreased DOX resistance. In addition, membrane transporter P-glycoprotein expression was found to be suppressed in a pathway downstream of PTEN [104]. Wang et al. found that miR-29a-3p mimics boosted irradiation resistance in HIEC, a normal human intestinal epithelial crypt cell line, while the inhibitors sensitized drug-resistant CRC cell lines. As was reported earlier in drug resistance, an attractive mechanism was that miR-29a-3p served as a suppressor of PTEN [96].

For radiotherapy resistance, Wang et al. proved that miR-29a-3p mimics increased the resistance to radiation in HIEC, the normal human intestinal epithelial crypt cell line, while the miR-29a-3p inhibitor could sensitize drug-resistant CRC cell lines. Interestingly, the underlying mechanism was that miR-29a-3p acted as a suppressor of PTEN as mentioned previously in drug resistance [108]. Taken together, despite the limited number of investigations, we noted that miR-29a-3p-targeted molecular therapy combined with radiotherapy and/or chemotherapy has the potential role to restore the treatment response and further improve the prognosis in advanced CRC patients with poor survival. Further studies should be conducted to find out more underlying mechanisms.

#### MiR-29a-3p-based drugs

Mounting data suggests that anticancer drugs can halt the formation and progression of CRC by controlling the dysregulation of miR-29a-3p (Fig. 3). In order to counteract colonic carcinogenesis brought on by TGF-β, Huang et al. found that the molecular mechanisms of Chinese herbal medicines, including berberine and evodiamine, were associated with the overexpression of epigenetic factors, particularly miR-29a-3p and its target genes DNMT3A/3B [101]. Additionally, the natural food additive inositol hexaphosphate (IP6) is recognized to have anti-CRC properties. Through using microarray analysis, Kapral et al. revealed that its anticancer mechanism is also connected to a notable decline in miR-29a-3p in vitro experiments [105]. According to Kim et al., Astaxanthin (ATX) inhibited the ability of CRC to invade and spread through the down-regulation of MCY and further inactivation of the miR-29a-3p/MMP2 and miR-200a/ZEB1 axis. MCY is an oncogenic transcription factor and interacts adversely with miR-29a-3p in PC and AML [59, 86]. Kim et al. found that MCY suppressed miR-29a-3p and miR-200a expression in CRC. As a result, the anti-metastasis impact of ATX was mediated by MCY inhibition, followed by the restoration of miR-29a-3p and miR-200a expression. Additionally, they indicated that miR-29a-3p inhibited MMP2 production, which hampered the EMT process in vitro. As an aside, approximately 55% of individuals with metastatic CRC had KRAS mutations [106]. Therefore, Parakrama et al. examined the molecular characteristics of patients with KRAS mutant CRC following reovirus treatment for the first time based on the anticancer activity of reovirus. Reovirus therapy for KAS-mutated metastatic CRC resulted in considerably decreased miR-29a-3p expression in peripheral blood mononuclear cells and exosomes compared to standard chemotherapy and anti-VEGF treatment with FOLFIRI and bevacizumab. Functionally, miR-29a-3p upregulated the production of IFN-γ, which in turn activated CD8 + T cells and triggered their cytotoxic potential [107]. Consequently, down-regulation of miR-29a-3p was recognized as a measure of reovirus therapeutic response. Taken together, more investigations on the link between miR-29a-3p and immune regulation in CRC are promising to help physicians make a better decision for customized therapy options.

## Conclusion and prospect

Notably, miR-29a-3p aberrant expression in CRC may serve as a diagnostic and prognostic biomarker. Furthermore, miR-29a-3p expression in serum, exocrine, saliva, and feces will have a wider clinical utility as a non-invasive biomarker for co-detection. However, the question of whether miR-29a-3p is an oncogene or a tumor suppressor gene, and if miR-29a-3p differs in different CRC conditions, remains unresolved. Given the evidence for miR-29a-3p in CRC, the biological processes of proliferation, invasion, metastasis, the EMT process are closely related; however, further in-depth research into the complex regulatory mechanisms and functions of this miRNA is still required, both in vivo and in vitro. We observe that miR-29a-3p in the tumor immune microenvironment mostly serves a pro-cancer role in other cancers, which needs to be further investigated in CRC. This is based on the more extensive regulation mechanisms of miR-29a-3p in different other malignancies. Further investigation of additional ceRNAs that target miR-29a-3p will further provide light on CRC biology. Last but not least, miR-29a-3p has also been linked to the efficacy of some well-known medications and the responsiveness of current anticancer therapies, demonstrating its enormous potential as a therapeutic target for CRC. In conclusion, miR-29a-3p is a prospective marker for molecular targeting or therapeutic responsiveness as part of a customized and precise treatment approach for CRC patients. It is not just a viable candidate marker for diagnosis and prognosis. Most significantly, future relevant preclinical studies and clinical trials still need to be conducted in order to achieve the transfer from miR-29a-3p research to clinical application.
